# Incidence, surgical eligibility and outcome of spontaneous intracerebral haemorrhage in Southwest Finland – A retrospective study

**DOI:** 10.1016/j.bas.2024.102914

**Published:** 2024-08-06

**Authors:** Sami Lehto, Antti Sajanti, Santtu Hellström, Fredrika Koskimäki, Abhinav Srinath, Carolyn Bennett, Julián Carrión-Penagos, Ying Cao, Miro Jänkälä, Romuald Girard, Jaakko Rinne, Melissa Rahi, Janne Koskimäki

**Affiliations:** a Neurocenter, Department of Neurosurgery, Turku University Hospital and University of Turku, Finland; b Medical Research Center, Research Unit of Clinical Medicine, University of Oulu, Department of Ophthalmology, Oulu University Hospital, Oulu, Finland; c Neurovascular Surgery Program, Section of Neurosurgery, The University of Chicago Medicine and Biological Sciences, Chicago, IL, USA; d Department of Neurology, University of Chicago Medicine and The University of Chicago, IL, USA; e Department of Radiation Oncology, Kansas University Medical Center, Kansas City, KS, USA; f Department of Neurosurgery, Oulu University Hospital and University of Oulu, Finland

**Keywords:** Intracerebral haemorrhage, ICH, Incidence, Surgery, Anticoagulants, Outcome

## Abstract

**Introduction:**

Spontaneous intracerebral haemorrhage (sICH) is a major cause of morbidity and mortality. Large-scale trials have shown neutral outcomes for surgical interventions. The recent trial suggested functional benefits from surgical intervention. Surgical treatment for sICH is likely increasing.

**Research question:**

To determine the incidence of sICH in Southwest Finland, standardized to the European population, and to identify the proportion of large sICH patients eligible for surgery based on previously published trial criteria. We also examined factors associated with outcomes, including the effects of anticoagulant and antithrombotic medications.

**Material and methods:**

A retrospective clinical study identified 596 ICH cases treated at Turku University Hospital (2018–2019), of which 286 were supratentorial sICHs. Variables were analysed using a *t*-test, chi-squared or Fisher's exact test. A multivariate logistic modelling was performed to evaluate outcome differences.

**Results:**

The sICH incidence was 29.9/100,000 persons per year, with the highest European population age and sex standardized rates in individuals over 80 years old (110/100,000 males, 142/100,000 females). The incidence of sICH patients meeting surgical criteria was 2.7/100,000 persons per year. Out of 286 patients, 26 were eligible for surgery and had unfavourable outcomes (p = 0.0049). Multivariate analysis indicated a significant decrease in favourable outcomes with warfarin (p = 0.016, OR 0.42) and direct-acting anticoagulants (DOACs) (p = 0.034, OR 0.38), while antithrombotic medications showed no significant effect.

**Discussion and conclusion:**

We identified comparable incidence of sICH as European average. A small proportion of sICH cases were identified to be candidates for surgical intervention. Anticoagulants were associated with increased risk of unfavourable outcomes.

## Highlights

• The incidence of spontaneous intracerebral haemorrhage in Southwest Finland was comparable to averages in Finland and Europe.
• Only a small proportion of spontaneous ICH patients met the criteria for possibly benefitting for surgical treatment.
• Anticoagulant medications significantly increased the risk of unfavourable outcomes.

## Abbreviations:

ADPR =: adenosine diphosphate receptor
CLEAR =: Clot lysis: Evaluating Accelerated Resolution of Intraventricular Haemorrhage
CT =: computer tomography
DNR =: do not resuscitate
DOAC =: direct oral anticoagulant
ENRICH =: Early Minimally Invasive Removal of Intracerebral Haemorrhage
EOT =: end-of-treatment
GCS =: Glasgow coma scale
ICH =: intracerebral haemorrhage
IVH =: intraventricular haemorrhage
MISTIE III =: Minimally Invasive Surgery with Thrombolysis in ICH
mRS =: modified Rankin scale
STICH =: Surgical Treatment for ICH

## 1 Introduction

The incidence of spontaneous intracerebral haemorrhage (sICH) in Europe varies but is generally reported to be around 25–30 cases per 100,000 person-years (Li et al., 2022; Wafa et al., 2024). This estimate can fluctuate based on specific regional studies and population demographics (Li et al., 2022; Wafa et al., 2024; van Asch et al., 2010; Krishnamurthi et al., 2020). The incidence of sICH that could benefit from surgical treatment is not widely reported due to the lack of definitive evidence and selection criteria supporting the benefits of surgical intervention.

There have been several randomised trials comparing surgical and conservative treatment of spontaneous supratentorial ICH. The surgical treatment for ICH (STICH I) trial compared early surgical evacuation of the haematoma to the initial conservative treatment (Mendelow et al., 2005). However, it did not show a benefit in its primary outcome: good recovery or moderate disability on the extended Glasgow coma scale 6 months after ictus (Jennett and Bond, 1975; Jennett et al., 1981). Following this study, a subgroup analysis identified some heterogeneity in response to surgery in the trial patients. Patients with deeper and inaccessible ICHs seemed to have a worse outcome following surgery, while patients with more prominent and easily accessible lobar haemorrhages without intraventricular haemorrhage (IVH) showed better outcomes. STICH II, a second randomised trial compared early surgery and initial conservative treatment in lobar ICH without IVH (Mendelow et al., 2013).

The analyses of the primary outcome did not show a difference between proportion of patients with an unfavourable outcome at 6 months between those who received early surgery and those with initial conservative treatment. These two trials focused on early surgery and choice of method was left to the operating surgeon. As a result, these trials evaluated mainly the effect of craniotomy.

Minimally invasive technique was trialled by the Minimally Invasive Surgery with Thrombolysis in ICH (MISTIE-III) randomised trial, which compared stereotactic catheter thrombolysis to a more conservative standard of care (Hanley et al., 2019). The results of MISTIE-III were also neutral with respect to its primary outcome. All three trials, while by a wide margin the most powerful trials to compare surgery to initial or solely conservative treatment, suffered from lack of power for subgroup analyses, which is necessary to evaluate the multidimensional aspects of surgical patient selection (Evaniew et al., 2016).

However, there have been some very promising developments in the wake of post-hoc analyses of MISTIE-III. Subsequent post-hoc subgroup analyses have suggested a benefit of surgical intervention with correct patient selection in MISTIE-III (Awad et al., 2019; Polster et al., 2021). Awad et al. (2019) found a correlation between good functional outcome, 0 to 3 on the modified Rankin scale (mRS) and achieving an end of treatment (EOT) haematoma volume of less than 15 ml in the surgical arm of MISTIE-III (Awad et al., 2019). Additionally, they observed a threshold effect with MISTIE cases per surgeon and per site, where no poor results with EOT volumes more than 30 ml were observed. Polster et al. (2021) reported a correlation between good functional outcome and a lower EOT ICH volume threshold of 28.8 ml, with an additional 8% improvement in likelihood to reach a good outcome when lowering the threshold to 15 ml (Polster et al., 2021).

These developments have shown promise. Recently published Early Minimally Invasive Removal of Intracerebral Haemorrhage (ENRICH) trial assessed whether early minimally invasive surgical removal of hematomas would improve outcomes compared to medical management in patients with sICH (Pradilla et al., 2024). At 180 days, the surgery group had a higher mean score on the utility-weighted modified Rankin scale (0.458 vs. 0.374), indicating better outcomes. The surgery group also had lower 30-day mortality (9.3% vs. 18.0%). Furthermore, several ongoing trials such as the Dutch ICH Surgery Trial (NCT05460793) and EVACUATE-RCT (NCT04434807) are investigating minimally invasive surgical techniques for sICH treatment and hopefully provide new treatment possibilities in future (Sondag et al., 2023).

Motivated by these recent findings, we aimed retrospectively characterize the incidence of sICH including patients that could potentially have been benefitted surgery according to recent evidence. We also aimed to evaluate the factors that are associated to outcome, including antithrombotic and anticoagulant medication usage. Understanding the specific patient cohort and emphasizing the nuances of surgical timing, precision, desired post-operative volume, and the proficiency of the operating team and institution are paramount for developing and adopting new treatment possibilities.

## 2 Materials and methods

### 2.1 Study design

**Fig. 1:**
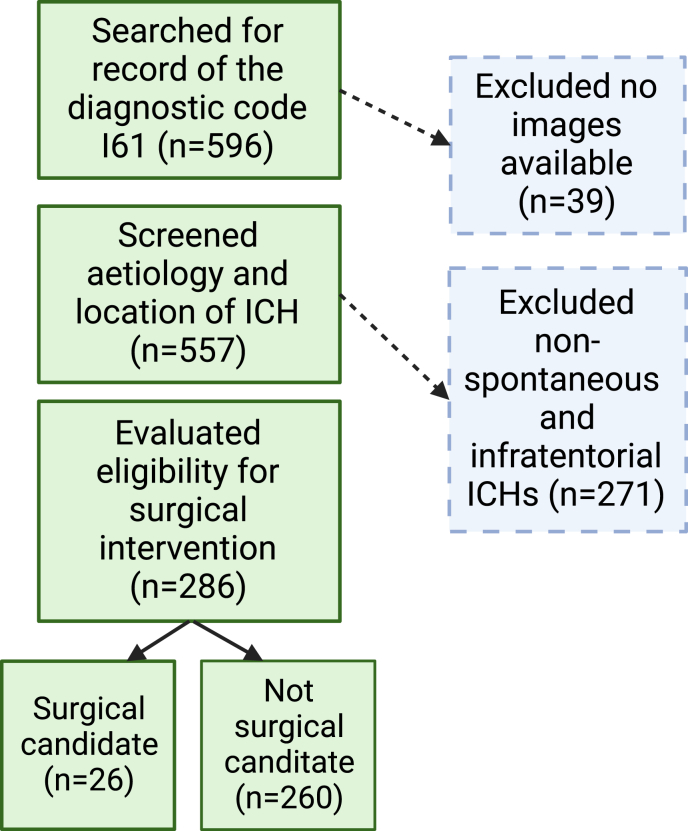
**Patient case selection.** 596 patient cases were identified having their first record of the International Classifications of Diseases 10 (ICD-10) code I61 during 2018–2019. Thirty-nine patient cases were excluded due to incomplete records. The computer tomography imaging taken at diagnosis and patient documentation were used to exclude non-spontaneous and infratentorial ICHs. The remaining 286 cases were then further analysed and compared to the trial eligibility criteria.

A search query was performed from electronic health records for patients who had received the ICD-10 diagnosis I61 (nontraumatic intracerebral haemorrhage) for the first time between 2018 and 2019 at the Turku University Hospital. In total 596 patient cases were identified. 39 cases were excluded due to lacking computer tomography (CT) imaging from initial diagnosis. A further 271 were excluded due to non-spontaneous or infratentorial origin (47 cerebellar, 24 basilar, 109 traumatic, 23 aneurysmatic, 19 secondary to ischaemic infarction, 14 neoplasia, 6 arteriovenous malformations, 4 dural arteriovenous fistulae, 2 cavernomas, 1 sinus thrombosis, 11 purely intraventricular haemorrhages without an intracerebral component, 3 non-aneurysmatic subarachnoid haemorrhages, 3 multiple ICHs, 1 extracranial haemorrhage, 4 patients less than 18 years old). A total of 286 cases were included for further data collection (Fig. 1). Of these, 26 patients would have been eligible according to the ENRICH trial criteria, while 261 would have been ineligible (Ratcliff et al., 2023). In our study, we retrospectively applied the same inclusion and exclusion criteria as the ENRICH trial, with the exception that we also considered haemorrhages larger than 80 ml as possible candidates for surgical intervention (Hanley et al., 2019; Awad et al., 2019). Shortly, for inclusion, individuals must present with (1) spontaneous supratentorial intracerebral haemorrhage of at least 30 ml, (2) demonstrate a Glasgow Coma Scale score of 14 or less, or a (3) National Institute of Health Stroke Scale score of 6 or more (Pradilla et al., 2024). The important exclusion criteria applied were uncorrectable coagulopathy or the need for long-term anticoagulation. (Pradilla et al., 2024). Full list of inclusion and exclusion criteria are previously published (Pradilla et al., 2024; Ratcliff et al., 2023).

Incidence estimates are based on the area, where Turku University Hospital is the closest hospital with on-call CT-imaging. This area, the region of Soutwest Finland has a population of 480,000 persons.

### 2.2 Factors associated with sICH

ICH volume was measured from the head-CT acquired at admission using the formula ABC/2, where A is the maximum haematoma diameter on the axial CT-slice with maximal haematoma area, B is the maximum diameter at a right angle to A, and C is the amount axial slices that show the haematoma multiplied by slice thickness (Kothari et al., 1996). This technique has been validated by Webb et al. (2015) based on data from MISTIE II, CLEAR-IVH and CLEAR III (Webb et al., 2015). They did not find a learning effect with ABC/2, although they noted that it tends to overestimate ICH volume compared to CT-based planimetry. Cases in which where the diagnosed ICH was small with indistinct borders and could not be measured with ABC/2 were not included. Midline shift was measured when possible as the greatest right-angle distance between the shifted apparent midline and the anatomical midline as estimated by the superior sagittal sinus posteriorly and crista galli anteriorly on axial slicing at mid-height of the 3rd ventricle.

Three-month mRS was assessed by a certified neurological nurse or neurologist (Broderick et al., 2017). Other variables gathered from included patients included: (1) age, (2) gender, (3) history of hypertension, (4) use of aspirin, (5) adenosine diphosphate receptor inhibitor (ADPR), (6) direct-acting oral anticoagulant (DOAC), (7) warfarin, (8) a prior “Do not resuscitate”-directive (DNR), (9) delay from ictus to diagnostic CT, (10) sICH location, (11) midline shift, (12) compression of basilar cisterns, and (13) associated intraventricular haemorrhage (IVH) on diagnostic CT, (14) hemiparesis, (15) dysarthria or aphasia, (16) neglect, (17) and GCS<8 at admission. Exclusion due to a strongly implied secondary cause was made based on CT-angiography, other features outlined in the radiology report or an evident history and/or clinical signs of trauma. Exclusion due to technical factors was mainly due to lack of access to CT-scan images or missing documentation of presentation on admission.

### 2.3 Statistical methods

For continuous variables a two-sample *t*-test was used for comparing means between mRS groups (favourable 0–3, unfavourable 4–6). If equal variance was not valid, Satterthwaite correction was applied. For categorical variables, Chi-square test or Fisher's exact test was used wherever appropriate for comparing proportions either between or among various categories. A multivariate logistic model on mRS was produced to evaluate if any difference existed between anticoagulant medications. SAS 9.4 (SAS Institute Inc., 2016; Cary, NC, US) was used in all statistical analyses. P < 0.05 was considered statistically significant. For incidence standardization, we used the 2013 European Standard Population, as defined by Eurostat, to calculate age and sex-standardized incidence rates.

## 3 Results

### 3.1 Study cohort

A total of 286 sICH cases were identified (Fig. 1). Demographic analysis (Table 1) showed an even proportion of women and men (152/286, 53.1% and 134/286, 46.9% respectively), with a mean age of 74.45 years (SD ± 44.8, [16–98]). Deep ICHs originating from deep structures such as the basal ganglia composed a slightly higher portion than lobar ICHs originating from subcortical white matter (142/286, 49.7% vs. 118/286, 41.3%), and a minority of the haematomas were holohemispheric haematomas. There was an associated IVH in 94/286 (32.9%) patients. A majority had a prior diagnosis of arterial hypertension (186/275, 66.2%). The use of antithrombotic or anticoagulative medications was relatively infrequent: aspirin and an ADPR inhibitor were used by 44/286 (15.4%) and 13/286 (4.5%) respectively, a direct oral anticoagulant (DOAC) and warfarin were used by 27/286 (9.4%) and 51/286 (17.8%), respectively. There were only a handful of combinations of an antithrombotic and anticoagulative medications: aspirin and an ADPR-inhibitor (4/286), aspirin and a DOAC (1/286), and ADPR-inhibitor and warfarin (2/286). There were no other combinations of the medications. On admission patients had hemiparesis in 172/237 (72.6%) cases, dysarthria or aphasia in 119/162 (73.5%) cases, a Glasgow coma scale of 8 or less in 50/118 (42.4%) cases, and hemispatial neglect in 81/128 (63.3%) cases. Delay from ictus to diagnostic computer tomography (CT)-imaging was on average 2.97 h (SD 2.38). A prior DNR – statement had been recorded with 21/286 (7.3%) patients.

**Table 1:** Demography and disease characteristics of intracerebral haemorrhage patients (N = 286).

Variable	Level	N = 286	%
Surgical candidate	No	260	90.9
	Yes	26	9.1
Gender	Female	152	53.1
	Male	134	46.9
ICH localization	Deep	142	49.7
	Lobar	118	41.3
	Holohemispheric	26	9.1
Basilar compression	Absent	243	85.0
	Present	43	15.0
Associated IVH_1_	Absent	192	67.1
	Present	94	32.9
Prior diagnosis of hypertension	No	93	33.8
	Yes	182	66.2
	Missing	11	–
Use of aspirin	No	242	84.6
	Yes	44	15.4
Use of APDR_2_-inhibitor	No	273	95.5
	Yes	13	4.5
Use of DOAC_3_	No	259	90.6
	Yes	27	9.4
Warfarin	No	235	82.2
	Yes	51	17.8
Hemiparesis on admission	Absent	65	27.4
	Present	172	72.6
	Missing	49	–
Dysarthria or aphasia on admission	Absent	43	26.5
	Present	119	73.5
	Missing	124	–
Neglect on admission	Absent	47	36.7
	Present	81	63.3
	Missing	168	–
GCS_4_ on admission	≥8	68	57.6
	<8	50	42.4
	Missing	168	–
Prior DNR_5_-order	Recorded	265	92.7
	Not recorded	21	7.3
mRS_6_ at 3 months, favourable vs. unfavourable	Favourable	116	46.8
	Unfavourable	132	53.2
	Missing	38	–
mRS_6_ at 3 months	0	10	4.0
	1	22	8.9
	2	37	14.9
	3	47	19.0
	4	38	15.3
	5	9	3.6
	6	85	34.3
	Missing	38	–
mRS_6_ at 3 months	Mean	3.37	
	Median	3	
	Minimum	0	
	Maximum	6	
	Missing	38	
Age, years	Mean	74.45	
	Median	76	
	Minimum	16	
	Maximum	98	
	Std deviation	44.8	
	Missing	0	
ICH volume, ml	Mean	30.86	
	Median	12.58	
	Minimum	0.02	
	Maximum	277	
	Std deviation	44.88	
	Missing	0	
Ictus to CT - delay, hour	Mean	2.97	
	Median	1.84	
	Minimum	0.25	
	Maximum	17.42	
	Std deviation	3.28	
	Missing	152	
Midline shift, mm	Mean	3.27	
	Median	0	
	Minimum	0	
	Maximum	35	
	Std deviation	5.48	
	Missing	0	

1IVH = intraventricular haemorrhage, _2_ADPR = adenosine diphosphate receptor, _3_DOAC = direct-acting oral anticoagulant, _4_GCS = Glasgow coma scale, _5_DNR = Do not resuscitate, _6_mRS = modified Rankin scale.

### 3.2 Incidence of sICH

**Fig. 2:**
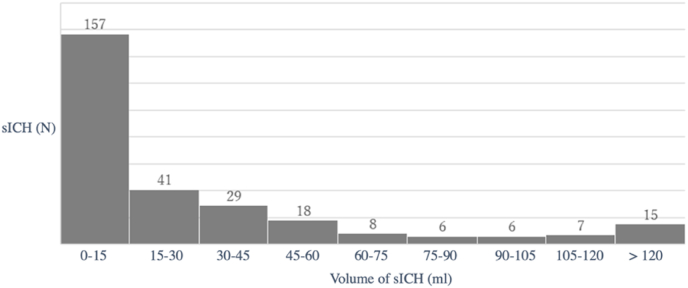
Histogram of spontaneous intracerebral haemorrhage (sICH) volumes (N = 286).

Two-hundred-sixty patients (90.9%) were surgery ineligible, while 26 (9.1%) were considered eligible. The total estimated incidence of ICH was 29.9 per 100,000 persons per year and the estimated incidence of surgery eligible ICH is 2.7 per 100,000 per year. Average ICH volume was 30.86 ml (SD ± 44.88) and median ICH volume was 12.58 ml (Table 1 and Fig. 2).

The incidence of sICH in Southwest Finland was also analysed and standardized to the European standard population with age and sex adjustment (Table 2). The standardized incidence rates for males were 12 per 100,000 in the 20–39 age group, 57 per 100,000 in the 40–59 age group, 31 per 100,000 in the 60–79 age group, and 110 per 100,000 in those over 80 years old. For females, the standardized rates were 16 per 100,000 in the 20–39 age group, 42 per 100,000 in the 40–59 age group, 35 per 100,000 in the 60–79 age group, and 142 per 100,000 in those over 80 years old.

**Table 2:** Spontaneous intracerebral haemorrhage incidences in Southwest Finland with age and sex standardization to the European standard population.

Age group (years)	sICH incidence males/100,000	sICH incidence females/100,000	Standardized sICH incidence for European standard population males/100,000	Standardized sICH incidence for European standard population females/100,000
**18**–**19**	0.1	0	NA	NA
**20**–**39**	0.3	0.4	1,2	1,6
**40**–**59**	1.5	1.0	5,7	4,2
**60**–**79**	6.2	7.0	31	35
**> 80**	5.5	7.0	110	142

NA = not applicable; sICH = spontaneous intracerebral haemorrhage.

### 3.3 Surgery eligible and ineligible cohorts

The demographic and disease variables were comparable between identified surgery eligible and ineligible sICH patients (Table 3). Fewer patients achieved a good functional outcome (mRS 0–3 at 3 months) among those eligible compared to ineligible (p = 0.0049, 4/22, 15.4% vs. 114/228, 49.1%). No patient in the surgery eligible cohort achieved an mRS of 0 or 1 compared to 32/228 (14.2%) in the ineligible cohort. ICHs were larger in the eligible cohort (p < 0.0001, 64.2 ± 38.4 ml vs. 27.5 ± 44.2 ml). While the surgery ineligible group contained some very large ICHs, an overwhelming majority of ICHs were ineligible due to ICH volume being <30 ml (n = 197/260, 75.8%) (Table 3). From those patients that were operable by size (>30 ml, n = 64), 27 patients were ineligible due to anticoagulation medication (n = 27/64, 42.1%). On admission hemiparesis was more common in the eligible cohort (p = 0.0055, 24/25, 96.0% vs. 148/212, 69.8%), as well as hemispatial neglect (p = 0.0150, 16/18, 88.9% vs. 64/109, 58.7%).

**Table 3:** Comparison between surgery eligible and ineligible cohorts.

	Eligible (n = 26)		Ineligible (n = 260)		p-value
	No.	(%)	No.	(%)	
Age, mean (SD), years	70.5	(16.3)	74.8	(13.6)	0.1287
Female	11	(57.7)	141	(54.2)	0.2456
Male	15	(42.3)	119	(45.8)	0.2456
History of hypertension	12	(50.0)	170	(67.7)	0.0794
Use of aspirin	7	(26.9)	37	(14.2)	0.0872
Use of ADPR-inhibitor_1_	3	(11.5)	10	(3.8)	0.0726
Use of DOAC_2_	0	(0.0)	27	(10.4)	0.0842
Use of warfarin	0	(0.0)	51	(19.6)	0.0127
DNR_3_ - order	0	(0.0)	21	(8.1)	0.1322
Ictus-to-CT delay, hours	2.4	(2.4)	3.0	(3.4)	0.4460
ICH volume, ml	64.2	(38.4)	27.5	(44.2)	**<0.0001**
ICH localization					0.2741
- deep	9	(34.6)	133	(51.2)	
- lobar	14	(53.8)	104	(40.0)	
- holohemispheric	3	(11.5)	23	(8.8)	
Midline shift, mm	4.7	(4.5)	3.1	(5.6)	0.1554
Compression of basilar cisterns	5	(19.23)	38	(14.73)	0.5301
Associated IVH_4_	12	(46.15)	82	(31.54)	0.1304
mRS_5_ at 3 months post ictus					**0.0049**
- 0–3 = favourable outcome	4	(15.4)	112	(49.6)	
- 4–6 = unfavourable outcome	18	(69.2)	114	(50.4)	
Symptoms at admission					
- Hemiparesis/plegia	25	(96.0)	148	(69.8)	**0.0055**
- Dysarthria/afasia	11	(78.6)	108	(73.0)	0.6502
- Neglect	16	(88.9)	64	(58.7)	**0.0150**
- GCS_6_ < 8	5	(35.7)	45	(43.3)	0.5912

1ADPR = adenosine diphosphate receptor, _2_DOAC = direct-acting oral anticoagulant, _3_DNR = Do not resuscitate, _4_IVH = intraventricular haemorrhage, _5_mRS = modified Rankin scale, _6_GCS = Glasgow coma scale.

### 3.4 Factors associated with outcome after sICH

In the total study cohort (n = 286), we identified several factors associated with a favourable outcome (mRS 0–3) (Table 4). Patients who achieved a favourable outcome were younger (p < 0.0001, 71.2 ± 13.5 years vs. 78.3 ± 13.3 years), fewer had prior DNR-orders (p = 0.0006, 2/116, 1.7% vs. 18/132, 13.6%), fewer met the criteria for surgery (p = 0.0049, 4/116, 3.4% vs. 18/132, 13.6%), they had smaller sICHs (p < 0.0001, 11.6 ± 13.4 ml vs. 52.1 ± 57.2 ml), they had a different distribution of sICH locations, they had a smaller midline shift (p < 0.0001, 0.9 ± 1.9 mm vs. 5.8 ± 6.9 mm), they initially presented less often with hemiparesis (p < 0.0001, 63/106, 59.4% vs. 87/98, 88.8%), or hemispatial neglect (p < 0.0001, 20/53, 37.7% vs. 50/60, 83.3%), and had a GCS of 7 points or less (p < 0.0001, 5/35, 14.3% vs. 43/70, 61.4%).

**Table 4:** Comparison of ICHs with favourable (modified Rankin Scale (mRS) 0–3) and unfavourable (mRS 4–6) outcomes.

	Total (n = 248)		Favourable (n = 116)		Unfavourable (n = 132)		p -value
	No.	(%)	No.	(%)	No.	(%)	
Age, years	75	(13.8)	71.2	(13.5)	78.3	(13.3)	**<0.0001**
Female	130	(52.4)	59	(50.9)	71	(53.8)	0.6453
Male	118	(47.6)	57	(49.1)	61	(46.2)	0.6453
History of hypertension	158	(66.4)	70	(61.9)	88	(70.4)	0.1680
Use of aspirin	36	(14.5)	14	(5.7)	22	(8.9)	0.3051
Use of ADPR_1_-inhibitor	10	(4.0)	4	(1.6)	6	(2.4)	0.6612
Use of DOAC_2_	25	(10.1)	8	(3.2)	17	(6.9)	0.1185
Use of warfarin	44	(17.7)	15	(6.1)	29	(11.7)	0.0630
DNR_3_-order	20	(8.1)	2	(1.7)	18	(13.6)	**0.0006**
Surgery eligible	22	(8.9)	4	(3.4)	18	(13.6)	**0.0049**
ICH_4_ volume, ml	33.1	(47.2)	11.6	(13.4)	52.1	(57.2)	**<0.0001**
ICH_4_ location							**<0.0001**
- deep	122	(49.2)	56	(22.6)	66	(26.6)	
- lobar	100	(40.3)	60	(24.2)	40	(16.1)	
- holohemispheric	26	(10.5)	0	(0.0)	26	(10.5)	
Ictus-to-CT_5_-delay, hours	2.8	(3.3)	3.4	(3.9)	2.3	(2.5)	0.0795
Midline shift, mm	3.5	(5.7)	0.9	(1.9)	5.8	(6.9)	**<0.0001**
Symptoms at admission							
- Hemiparesis/plegia	150	(73.5)	63	(59.4)	87	(88.8)	**<0.0001**
- Dysarthria/afasia	107	(74.8)	54	(72.0)	53	(77.9)	0.4136
- Neglect	70	(61.9)	20	(37.7)	50	(83.3)	**<0.0001**
- GCS_6_ < 8	48	(45.7)	5	(14.3)	43	(61.4)	**<0.0001**

1ADPR = adenosine diphosphate receptor, _2_DOAC = direct-acting oral anticoagulant, _3_DNR = Do not resuscitate, _4_ICH = intracerebral haematoma, _5_CT = computer tomography, _6_GCS = Glasgow coma scale.

None of the recorded antithrombotic or anticoagulating medications differed statistically significantly, but warfarin and DOAC gave a signal toward significance (p = 0.063 and p = 0.1185 respectively). We performed multivariate analysis to further study the effect of antithrombotic and anticoagulative medications on outcome. There was a statistically significant decrease in likelihood of achieving a favourable outcome with the use of warfarin (p = 0.016, OR 0.42, 95% CI [0.21–0.85]) or a DOAC (p = 0.034, OR 0.38, 95% CI [0.15–0.93]) (Table 5). No significant effect was observed with the use of antithrombotic medications.

**Table 5:** Multivariate logistic regression analysis of antithrombotic and anticoagulant medications association with outcome in spontaneous intracerebral haemorrhage patients. Favourable outcome modified Rankin Scale (mRS) 0–3, Unfavourable outcome mRS 4–6.

Medication	Status	Odds Ratio (95% CI)	OR p-value
Warfarin	Yes	0.42 (0.21–0.85) ref	**0.016**
	No		
DOAC	Yes	0.38 (0.15–0.93) ref	**0.034**
	No		
Aspirin	Yes	0.52 (0.25–1.09) ref	0.084
	No		
ADPR-inhibitor	Yes	0.71 (0.19–2.65) ref	0.611
	No		

Number of observations in the data set = 286.

Ref = reference, DOAC = direct-acting oral anticoagulant, ADPR = adenosin diphosphate receptor.

## 4 Discussion

This retrospective study at Turku University Hospital aimed to determine the incidence of spontaneous intracerebral haemorrhage (sICH) in Southwest Finland, standardized to the European population, and to assess the proportion of patients eligible for surgical intervention. We analysed 286 cases of supratentorial sICH from 2018 to 2019, identifying 26 patients meeting surgical criteria. the study identified the incidence of sICH and sICHs that could potentially benefit for surgery. We also showed that warfarin and DOACs were associated with poorer outcomes.

We observed an incidence of 29.9 per 100,000 persons, while Feigin et al. (2021) reported a global incidence of 41.81 per 100,000 persons, and a previous estimate of the incidence in Finland has been 15 per 100,000 persons and incidences reported in past have varied between 15 and 35 per 100,000 (GBD, 2019 Stroke Collaborators, 2021; Fogelholm et al., 1992; Huhtakangas et al., 2011; Sivenius et al., 2004). Our estimate was on the higher end of ICH rates reported in Finland and in industrialised countries, however, when age and sex standardization to European population was performed, incidence in Southwest Finland was slightly lower than recently published in Europe (Wafa et al., 2024). It should be noted the hospital studied is a tertiary care centre with neurointensive care and neurosurgical units and covers almost 1 million persons requiring advanced neurointensive care measures or neurosurgical intervention. While hospital transfers thereby increase proportional incidence and bias our findings toward patients with larger and more complicated ICHs, the vast majority of included cases were not hospital transfers.

### 4.1 Surgically eligible and ineligible cohorts

Going by the very strict selection criteria for trial the incidence of potentially operable ICH is very low. However, even with this lower bound estimate this results in an incidence of 13 patients per year in a specialist centre with a total intake area of a million people. According to post-hoc analyses by Awad et al. (2019) there were no cases of poor performance (i.e. defined as end-of-therapy haematoma volume more than 30 ml) after 4 prior cases per surgeon and 7 prior cases per centre (Awad et al., 2019). As we observed a yearly incidence of 13 cases, surgical treatment of ICH could be feasibly implemented and maintain the surgical competence of three specialised surgeons. While the timing of the surgery remains a debate and logically faster would be better, post-hoc analyses by Polster et al. (2021) suggest a very wide therapeutic window up to potentially 47 h before an increase in mortality or 62 h until a decrease in achieving a favourable functional outcome (Polster et al., 2021). According to this piece of evidence, operations could be even concentrated to a small number of specialised centres and during typical daytime working hours, enabling a centre to maintain adequate surgical skill with few specialised surgeons. However, sufficient evidence is lacking drawing definitive conclusions.

### 4.2 Factors associated with outcome

ICH location was statistically significantly correlated with outcome at 3 months. This is likely mainly due to inclusion of holohemispheric as an independent location category (Table 3). There was a statistically significant difference in outcomes with ICH volume, and likely due to this there were also significant difference in midline shift, hemiparesis, neglect and GCS <8. However, there was no difference in dysarthria and aphasia, which may be due poor documentation.

Anticoagulant associated sICHs has been increasing in absolute incidence and proportion of all ICHs, even while there has been increasing preference for DOACs, which appear to be less associated with ICH than warfarin (Flaherty et al., 2007; Ruff et al., 2014). We also found a statistically significant decrease in likelihood to achieve a favourable outcome with use of warfarin or DOAC. American Heart Association guidelines recommend reversal of anticoagulation, which would also be necessary for surgery (Cervera et al., 2012; Greenberg et al., 2022). But as ENRICH excluded patients requiring long-term anticoagulation the role of surgery in anticoagulant-associated ICH remains unclear (Wafa et al., 2024; Pradilla et al., 2024).

### 4.3 Limitations

This was a retrospective study and thus suffers from missing data. Several demographic factors, such as smoking, and alcohol use could not be included due to their poor and infrequent documentation. The surgically eligible population was small (n = 26), which may introduce bias into the statistical analyses. Additionally, the surgical eligibility of patients was determined retrospectively, whereas real-world surgical decisions require multifaceted information, including input from the patient or legal representative. Despite these limitations, we believe this study reflects the potential patient population that could have benefitted from surgical treatment. Our estimate on the incidence of sICH assumes that all sICH cases from Southwest Finland were treated in Turku University Hospital, however there were also large ICHs, that came from neighbouring regions and were transferred to Turku University Hospital for tertiary care. These ICHs were larger or otherwise complicated and have thereby biased to larger haematomas. Cases of very small ICHs of uncertain clinical significance may be underreported. Use of aspirin in Finland could be underreported, even for secondary prevention of cardiovascular event it is often used without a prescription and therefore it is often missing from patient documentation despite daily use (Hellman et al., 2020). It should be noted that, as DOACs are far more popular in Finland and most patients who were initially started on warfarin have since been switched onto a DOAC. Therefore, patients taking warfarin may be taking it, because DOACs are contra-indicated for them, i.e. a mechanical heart valve. So, use of warfarin may also be a proxy for several unrecorded medical conditions.

## 5 Conclusion

This study provides valuable insights into the incidence, surgical eligibility, and outcomes of sICH in Southwest Finland. We found a similar incidence of sICH compared to European average. Only a small proportion of sICH cases met the criteria for surgical intervention. Our findings also corroborated previous studies by demonstrating that the use of warfarin and DOACs significantly increase the risk of unfavourable outcomes. Given the substantial morbidity associated with sICH, further clinical trials and studies are essential to refine treatment strategies and improve patient quality of life.

## Funding

No specific funding was used in this study.

## Availability of data and material

The anonymized data from this study can be made available upon request to qualified researchers who have obtained appropriate institutional review board (IRB) approval. Requests should be directed to the corresponding author (JK).

## Code availability

Not applicable.

## Authors' contributions

The study was conceptualized and designed by J.K. Data collection was carried out by S.L. Statistical analyses were conducted by Y.C. (biostatistician), J.K., S.L and F.K. The results were interpreted, and the initial manuscript was drafted by S.L., A.S., S.H., A.Sr., C.B., F.K., and J.K. The manuscript was critically reviewed, edited, and revised by J.C., M.J., M.R., J.R. and R.G. All authors have read and approved the final version of the manuscript for submission.

## Ethics approval

This study was approved by the Institutional Review Board of the Turku University Hospital (T52/2021). All procedures performed in studies involving human participants were in accordance with the ethical standards of the Institutional Review Board of the Turku University Hospital and with the 1964 Helsinki declaration and its later amendments or comparable ethical standards.

## Consent to participate

Due to its nature as a retrospective registry-based study, patient consent was not required and was not sought.

## Consent for publication

In this retrospective study, all data presented has been fully anonymized. No identifiable information or specific patient data is included. Given the retrospective nature of the analysis and the adherence to data privacy standards, no individual patient consent was required for publishing this study.

## Declaration of competing interest

The authors declare that they have no known competing financial interests or personal relationships that could have appeared to influence the work reported in this paper.
